# Post-COVID-19 Physical Activity and Symptom Burden in Patients with Asthma and COPD Compared with Individuals Without Chronic Disease: A Multicenter Cross-Sectional Study

**DOI:** 10.3390/diagnostics16040604

**Published:** 2026-02-19

**Authors:** Neslihan Köse Kabil, Dilek Karadoğan, Tahsin Gökhan Telatar, İlknur Kaya, Merve Yumrukuz Şenel, Merve Erçelik, Aycan Yüksel, Feride Marim, Metin Akgün

**Affiliations:** 1 Department of Chest Diseases, School of Medicine, Yalova University, Yalova 77300, Türkiye; 2 Department of Chest Diseases, School of Medicine, Recep Tayyip Erdoğan University, Rize 53020, Türkiye; cakmakcidilek@yahoo.com; 3 Department of Public Health, School of Medicine, Recep Tayyip Erdoğan University, Rize 53020, Türkiye; gokhantelatar@gmail.com; 4 Department of Chest Diseases, School of Medicine, Kutahya Health Sciences University, Kütahya 43050, Türkiye; ilknur_can89@hotmail.com (İ.K.); feridemarim@hotmail.com (F.M.); 5 Department of Chest Diseases, School of Medicine, Balıkesir University, Balıkesir 10145, Türkiye; mryumrukuz@gmail.com; 6 Department of Chest Diseases, School of Medicine, Suleyman Demirel University, Isparta 32260, Türkiye; evrem-33@hotmail.com; 7 Department of Chest Diseases, School of Medicine, TOBB University Of Economics and Technology, Ankara 06560, Türkiye; aycnakbas@hotmail.com; 8 Department of Chest Diseases, School of Medicine, Ağrı İbrahim Çeçen University, Ağrı 04100, Türkiye; akgunm@gmail.com

**Keywords:** COVID-19, asthma, chronic obstructive pulmonary disease, physical activity, post-COVID-19 symptoms, lifestyle changes

## Abstract

**Background/Objectives:** The COVID-19 pandemic led to profound lifestyle changes and long-term functional consequences, particularly among individuals with chronic respiratory diseases. Patients with asthma and chronic obstructive pulmonary disease (COPD) may be especially vulnerable to reductions in physical activity and persistent post-COVID-19 symptoms. This study aimed to compare lifestyle characteristics, physical activity levels, and post-COVID-19 symptom persistence in patients with asthma and COPD with those of individuals without chronic disease in the post-pandemic period. **Methods:** This national, multicenter, cross-sectional study was conducted in 2022 at five pulmonary outpatient clinics. Participants were categorized into three groups: asthma (*n* = 165), COPD (*n* = 82), and individuals without chronic disease (*n* = 431). Demographic and clinical data were collected through face-to-face structured interviews. Physical activity levels were assessed using the short form of the International Physical Activity Questionnaire and expressed as metabolic equivalent of task (MET) scores before and after the pandemic. Dyspnea severity was evaluated using the modified Medical Research Council scale. COVID-19 history, disease severity, and persistent symptoms were recorded. **Results:** A total of 678 participants were included. The median age was highest in the COPD group (68 (61–74) years), followed by the asthma group (54 (42–64) years) and individuals without chronic disease (38 (27–50) years). Female sex predominated among patients with asthma (77%), whereas male sex was more frequent in the COPD group (83%); sex distribution was similar among individuals without chronic disease (51% female). Across all groups, post-COVID-19 symptoms—including dyspnea, cough, fatigue, and myalgia—persisted for at least six months after infection. Physical activity levels, assessed by metabolic equivalent of task (MET) scores, declined significantly in the post-pandemic period in all groups, with the lowest levels observed in patients with COPD. COVID-19 severity and hospitalization rates were higher in patients with COPD, while intensive care unit admission rates were comparable between patients with asthma and individuals without chronic disease. **Conclusions:** In the post-pandemic period, physical activity levels declined markedly, and lifestyle changes were negatively affected in patients with asthma and COPD. Post-COVID-19 symptoms persisted longer than expected even in those without chronic disease. Therefore, individualized home-based exercise programs and psychosocial support should be considered to improve physical activity and quality of life, particularly in patients with chronic respiratory diseases, while preventive strategies should also be implemented at the population level.

## 1. Introduction

In March 2020, the World Health Organization (WHO) emphasized the concept of “isolation” as a key strategy for controlling the course of the COVID-19 pandemic [1]. Owing to the increased risk of mortality and morbidity, isolation measures were implemented earlier and more strictly in individuals aged 65 years and older and in those with comorbid conditions. Despite these measures, more than six million deaths related to COVID-19 were reported worldwide by June 2023 [2,3]. COVID-19 infection has been associated with pneumonia and respiratory failure, leading to intensive care unit admissions, the need for mechanical ventilation, and the development of acute respiratory distress syndrome (ARDS). Because the disease primarily affects the respiratory system and is associated with high mortality, levels of fear and anxiety increased particularly among patients with chronic respiratory diseases, such as asthma and chronic obstructive pulmonary disease (COPD).

The clinical course of COVID-19 in patients with asthma remains controversial and has not been fully clarified. While some studies suggest that COVID-19 infection increases hospitalization rates in patients with asthma, they do not report a corresponding increase in severe outcomes or mortality [3]. In a large COVID-19 cohort including 3731 patients with asthma, rates of mechanical ventilation and mortality were not higher compared with other comorbid conditions. Advanced age, increased body mass index (BMI), sex, lack of vaccination, hypertension, and chronic kidney disease were identified as factors associated with an increased risk of mechanical ventilation in patients with asthma. Moreover, treatment duration was found to be shorter in patients with asthma compared with those without asthma or COPD [4]. These findings are supported by studies suggesting that inhaled corticosteroids may reduce the severity of COVID-19 infection [5,6]. Although asthma has been reported not to be a risk factor for severe COVID-19 in several countries [7,8], patients with recent asthma exacerbations requiring hospitalization have been shown to experience a more severe course of COVID-19. Consequently, the coexistence of asthma and COVID-19 is considered clinically heterogeneous [9].

In patients with COPD and COVID-19 infection, male sex, advanced age, and smoking prevalence have been reported to be higher, while BMI tends to be lower compared with patients with asthma. Most patients with COPD were vaccinated, and treatment duration was longer than in patients with asthma. Patients with COPD and those with complicated asthma experienced higher rates of mechanical ventilation and mortality related to COVID-19 [4]. Although inhaled corticosteroids (ICS) have been suggested to increase the risk of pneumonia in patients with asthma and COPD [10], an opposite pattern emerged during the COVID-19 pandemic. In a subgroup analysis of patients with COPD, prior use of ICS was associated with a lower mortality risk compared with non-users [11]. In addition, ICS use was shown to facilitate post-COVID-19 symptom control and reduce hospitalization rates [12]. Pulse steroid therapy used in the management of complicated COVID-19 pneumonia further drew attention to the role of corticosteroids. Nevertheless, evidence regarding outcomes in patients using ICS prior to COVID-19 infection remains inconsistent.

There are regional and ethnic variations in the impact of COVID-19 infection on asthma and COPD and in the persistence of post-COVID-19 symptoms. 

The aim of this study was to evaluate disease course in patients with asthma and COPD with and without a history of COVID-19 infection, to assess changes in physical activity levels, to determine the persistence of post-COVID-19 symptoms and identify the groups in which these symptoms persist, to compare these findings with individuals without chronic disease, and to highlight considerations for preventive strategies in potential future pandemics.

## 2. Materials and Methods

### 2.1. Study Design and Setting

This national, multicenter, cross-sectional study was conducted between 1 June 2022 and 1 September 2022. A total of 678 participants were consecutively recruited from the pulmonary outpatient clinics of five centers located in Yalova, Kütahya, Ankara, Afyon, and Balıkesir.

### 2.2. Participants and Eligibility Criteria

Individuals aged 18 years and older who presented to the pulmonary outpatient clinics during the study period were screened for eligibility. Patients were included if they met one of the following criteria: (i) a diagnosis of asthma, (ii) a diagnosis of chronic obstructive pulmonary disease (COPD), or (iii) absence of any chronic disease. Participants with a history of COVID-19 infection were required to have polymerase chain reaction (PCR)-confirmed infection with at least six months elapsed since PCR positivity. Individuals without a history of COVID-19 infection were also eligible for inclusion.

Exclusion criteria were as follows: conditions limiting physical mobility within the preceding one month (including recent intensive care unit admission, orthopedic surgery, intracranial surgery, abdominal or thoracic surgery), recent cerebrovascular accident or myocardial infarction, a history of intracranial or intra-abdominal hemorrhage, pregnancy, lack of informed consent, being within the first six months following PCR-confirmed COVID-19 infection, and the presence of chronic comorbidities other than asthma or COPD.

### 2.3. Data Collection

All eligible participants underwent face-to-face structured interviews conducted by trained investigators. Demographic and clinical data were recorded, including age, sex, caregiving responsibility (same household or different household), occupation (retired, healthcare worker, engineer, public sector employee, worker, private sector employee), current employment status, income level (below minimum wage, minimum wage or above, twofold minimum wage or above, threefold minimum wage or above), changes in working environment (home office, office-based, hybrid), place of residence (urban or rural), body mass index (BMI), smoking and alcohol consumption history, history of chronic disease, use of psychological support services, and sleep patterns.

Symptom burden was defined as the presence and persistence of symptoms such as dyspnea, cough, fatigue and myalgia lasting at least six months after COVID-19 infection. Dyspnea severity was assessed using the modified Medical Research Council (mMRC) scale for the pre-pandemic and post-pandemic periods; in participants with a history of COVID-19 infection, mMRC scores during the acute disease period were also recorded. For individuals with COVID-19 infection, time since infection, disease severity, and the presence of persistent symptoms were documented.

### 2.4. Assessment of Physical Activity

Physical activity levels were assessed using the short form of the International Physical Activity Questionnaire (IPAQ), consisting of seven items. Physical activity intensity was quantified using metabolic equivalent of task (MET) values, defined as 1.5 METs for sitting, 3.3 METs for walking, 4.0 METs for moderate-intensity physical activity, and 8.0 METs for vigorous-intensity physical activity. Daily and weekly physical activity levels were calculated accordingly, and total MET-minutes per week were derived for each participant [13].

### 2.5. Statistical Analysis

Statistical analyses were performed using IBM SPSS 23.0 (Statistical Package for Social Sciences) Statistics for Windows (IBM Corp., Armonk, NY, USA). Normality of continuous variables was assessed using the Shapiro–Wilk test and visual inspection of histograms and Q–Q plots. As MET scores were not normally distributed, continuous variables are presented as mean ± standard deviation for descriptive purposes, and non-parametric tests were used for comparisons.

Within-group comparisons of pre-pandemic and post-pandemic MET scores were performed using the Wilcoxon signed-rank test. Between-group comparisons (asthma, COPD, and individuals without chronic disease) were conducted using the Kruskal–Wallis test with Dunn–Bonferroni post hoc correction where appropriate. Categorical variables were compared using the chi-square test.

The primary analyses focused on within-subject comparisons of pre-pandemic and post-pandemic MET scores. Between-group comparisons were considered secondary analyses.

To address potential confounding in between-group comparisons due to differences in age and sex, multivariable regression analyses were additionally performed. Disease group was included as the main independent variable, with age and sex as covariates. A two-sided *p*-value < 0.05 was considered statistically significant.

## 3. Results

A total of 678 participants were included in the study, consisting of 165 patients with asthma, 82 patients with chronic obstructive pulmonary disease (COPD), and 431 individuals without chronic disease. The mean age was significantly higher in the COPD group (68 (61–74) years) compared with the asthma group (54 (42–64) years) and individuals without chronic disease (38 (27–50) years) (*p* < 0.001). Body mass index (BMI) was highest among patients with asthma (28 ± 5 kg/m^2^). Female sex predominated in the asthma group (77%), whereas male sex was more common in the COPD group (83%). Sex distribution was comparable in individuals without chronic disease (51% female). Living alone was uncommon in all groups, and residence in urban areas was more frequent across the study population (*p* < 0.001).

The proportion of retired participants was higher in the asthma (63%) and COPD (62%) groups compared with individuals without chronic disease (32%), while active employment was more common among those without chronic disease (*p* < 0.001). The use of psychiatric support services was low in all groups (*p* < 0.001). Although no significant overall change in tobacco use was observed during the pandemic period, among participants reporting a change, smoking reduction was more frequently observed across all groups. Alcohol consumption was generally low but was significantly more prevalent in the group without chronic disease (*p* < 0.001) (Table 1 and Table 2).

**Figure 1:**
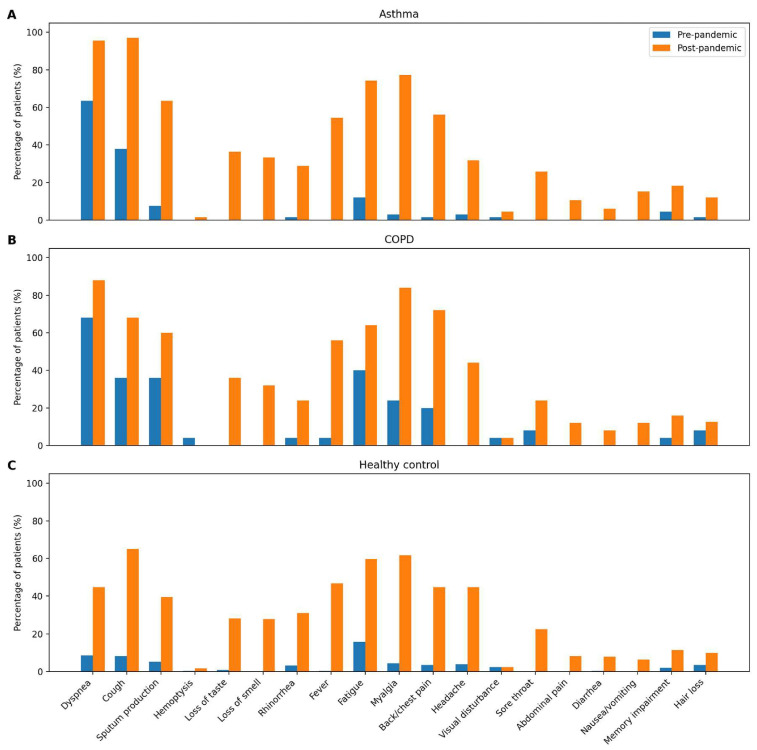
Changes in symptom prevalence before and after the COVID-19 pandemic among patients with asthma (**A**), chronic obstructive pulmonary disease (COPD) (**B**), and individuals without chronic disease (**C**). Values represent the percentage of patients reporting each symptom in the pre-pandemic and post-pandemic periods.

A history of COVID-19 infection was identified in 66 patients with asthma, 25 patients with COPD, and 255 individuals without chronic disease. Persistent post-COVID-19 symptoms—including dyspnea, cough, sputum production, loss of smell and taste, myalgia, headache, forgetfulness, and hair loss—were frequently reported in all groups, despite at least six months having elapsed since infection (Figure 1).

Before the pandemic, total metabolic equivalent of task (MET) scores were highest in individuals without chronic disease, followed by patients with asthma and those with COPD (*p* < 0.001). Pre-pandemic sitting MET scores were significantly higher in the COPD group than in the other groups. After the pandemic, moderate intensity MET scores were higher in patients with asthma compared with those with COPD, while walking MET scores were highest among individuals without chronic disease. Post-pandemic sitting MET scores remained significantly higher in the COPD group. Post-pandemic total MET scores were significantly higher in patients with asthma and individuals without chronic disease compared with patients with COPD (Table 3).

Across all groups, the proportion of participants classified as Category 1 (inactive) was high before the pandemic and increased further in the post-pandemic period (*p* < 0.001). Total MET scores decreased significantly after the pandemic in all groups (*p* < 0.001). No significant differences were observed in modified Medical Research Council (mMRC) dyspnea scores during the pandemic period among the study groups (Table 4).

Regarding COVID-19 severity, 69.7% of patients with asthma were managed as outpatients, 28.8% required hospitalization, and 1.5% required intensive care unit (ICU) admission. Among patients with COPD, 57.7% were treated as outpatients, 34.6% required hospitalization, and 7.7% were admitted to the ICU. In contrast, 88.6% of individuals without chronic disease were managed as outpatients, 10.2% were hospitalized, and 1.2% required ICU admission. A significant association was observed between COPD and greater COVID-19 severity (*p* < 0.05).

COVID-19-related death within the family was reported by 9.8% of patients with asthma, 4.9% of patients with COPD, and 3.5% of individuals without chronic disease, with a statistically significant difference between groups (*p* < 0.05) (Table 5).

## 4. Discussion

This study compared lifestyle characteristics and physical activity levels in patients with asthma and chronic obstructive pulmonary disease (COPD) and those without chronic disease during the post-COVID-19 period. COPD patients were older, while asthma patients had a higher body mass index, and smoking rates decreased in all groups during the pandemic. Following the pandemic, physical activity levels decreased significantly across all groups, with a greater decline observed in asthma and COPD patients, accompanied by an increase in sedentary behavior. Post-COVID-19 symptoms were more pronounced in individuals with chronic respiratory disease, but the persistence of symptoms was also evident in individuals without chronic disease. Hospitalization and intensive care unit admission rates were higher in COPD patients, reflecting the greater severity of COVID-19, while intensive care unit admission rates were similar between asthma patients and individuals without chronic disease.

The prevalence of asthma has continued to increase following the COVID-19 pandemic, and the World Health Organization has projected that the global number of individuals with asthma will reach 400 million by 2025 [14]. Although asthma is not typically associated with high mortality, inadequate disease control is linked to reduced quality of life and increased healthcare utilization. COVID-19 infection has emerged as a new challenge for asthma control, particularly due to seasonal exacerbations and symptom overlap [15]. COPD remains a leading cause of morbidity and mortality worldwide and is the third most common cause of death in Türkiye. Infectious triggers further complicate disease control in COPD. Similar to previous studies involving COPD patients comprising cohorts of over 200 individuals, in our study, male gender, advanced age, and unemployment were more prevalent in the COPD group, while a decrease in daily activities and increased fear of death were reported more frequently and extensively compared to the pre-pandemic period [16,17]. In a study including 22 patients with asthma, physical activity levels were lower and sitting time and sleep disturbances were more frequent compared with healthy controls, whereas stress and fear levels were comparable [18]. In the present study, female sex and higher BMI were more common among patients with asthma. The use of psychological support services was similar between patients with asthma and healthy individuals but was more frequent among patients with COPD. Difficulties initiating and maintaining sleep were more prevalent in patients with asthma and COPD than in healthy participants. Although numerous studies, consistent with our findings, have reported a reduction in smoking during the pandemic [19], some reports suggest that economic stress and prolonged isolation may have contributed to an increase in first-time smoking initiation among individuals aged 30 years and older [20].

In a large cohort study conducted in the United Kingdom involving 8.3 million individuals, hospitalization rates were higher among patients with asthma; however, no significant association with mortality was observed except in cases of severe asthma and COPD [21,22]. Several studies have suggested that COVID-19 infection does not precipitate severe asthma exacerbations and does not increase intensive care unit admissions among patients with asthma, possibly due to the inhibitory effects of asthma medications on viral replication [23,24]. In a study conducted in Mexico, the prevalence of asthma was low among hospitalized COVID-19 patients [25]. The similar intensive care unit admission rates observed between patients with asthma and individuals without chronic disease in our study support these findings. The higher rate of COVID-19-related family loss among patients with asthma was again notable. During the pandemic, hospital admissions among patients with COPD were reported to be lower than in the pre-pandemic period, potentially due to reduced air pollution and enhanced public health measures [26]. In contrast, our study demonstrates higher rates of hospitalization and intensive care unit admission among COPD patients, highlighting that this group is more vulnerable when infected with COVID-19. Although some studies argue that the prevalence of asthma and COPD varies by region following COVID-19 infection [27], there is clear evidence independent of region that it increases the risk of mortality and morbidity for COPD. Persistent post-COVID-19 symptoms such as dyspnea, cough, fatigue, and impaired ability to perform daily activities have been reported to last longer than one month and may become chronic in individuals with COPD [28]. Increases in COPD Assessment Test (CAT) scores and deterioration in sleep quality have also been documented after the pandemic [29]. Among patients with asthma, post-COVID-19 dyspnea has been reported in up to 75% during exertion and in 34% at rest, with symptoms persisting for at least 12 weeks [30]. In this study, it was found that despite using inhaler treatment more regularly after COVID-19 infection, asthma and COPD patients experienced an increase in symptoms and continued symptoms for at least six months after the infection, compared to before the pandemic. Post-COVID-19 symptoms persisted for at least six months not only in patients with asthma and COPD, but also in individuals without chronic disease. This indicates that the long-term symptom burden is not limited to those with pre-existing respiratory conditions and demonstrates that the infection persists not only during the acute phase but also in the chronic phase.

Public health measures such as the closure of sports facilities and restrictions on outdoor activities led to reductions in physical activity levels even among healthy populations. Decreased physical activity has been documented in physiotherapy students during the pandemic [31]. Reduced physical activity may adversely affect disease prognosis and the management of comorbidities and is associated with increased levels of anxiety and depression [32,33]. Consistent with these observations, our study has shown that there has been a significant decrease in physical activity compared to pre-pandemic levels in asthma and COPD patients as well as in healthy individuals, and that inactivity has increased across all groups. Physical activity has decreased compared to pre-pandemic levels in patients with post-COVID-19 symptoms, and physical activity has also decreased in all groups who did not contract the disease after the pandemic. Therefore, during periods such as a pandemic, physical fitness must be improved in order to cope with morbidity, alongside measures such as disease control and isolation. The persistence of post-COVID-19 symptoms may also be related to physical activity limitations.

This study has several limitations. Physical activity and lifestyle data were collected using self-reported questionnaires, which may be subject to recall bias. However, the structured and specific nature of the questions likely minimized this potential bias. Due to the focus on post-pandemic lifestyle changes, parameters such as disease control levels (e.g., ACT, CAT, GOLD stage) were not systematically collected. However, although it is unclear what treatment patients were receiving prior to COVID-19, their symptoms worsened despite an increase in inhaler treatment after infection. In other words, despite receiving more regular treatment, their post-COVID-19 symptoms increased. In addition, differences in age distribution across groups may influence the extent to which individuals were affected by pandemic-related restrictions, limiting the generalizability of the findings. However, as each group was also compared separately before and after the pandemic, the distribution within the groups was not affected by age differences in terms of symptoms or physical activity levels. Nevertheless, the multicenter design, large sample size, and comparative evaluation of individuals with and without chronic respiratory disease represent important strengths of the study. As tests such as the Six-Minute Walk Test (6MWT) or Cardiopulmonary Exercise Testing (CPET) were not available prior to the pandemic, the International Physical Activity Questionnaire (IPAQ) short form was used as an important standardized test to ensure patients could recall and answer the questions accurately without becoming bored.

## 5. Conclusions

During pandemic and endemic periods, the control of chronic diseases becomes more challenging, particularly for respiratory conditions associated with increased morbidity and mortality, where social isolation, fear of death, and anxiety contribute to lifestyle and functional decline. In the post-COVID-19 period, patients with asthma and chronic obstructive pulmonary disease (COPD) have shown significant decreases in physical activity, an increase in post-COVID-19 symptoms, psychological distress, and negative lifestyle changes. Even in healthy individuals, the chronicity of symptoms, along with measures leading to isolation and loneliness, has highlighted the need for different interventions. Targeted post-pandemic interventions, such as personalized rehabilitation, home exercise programs, and psychosocial support, should be increased to improve quality of life and disease management. Importantly, the persistence of post-COVID-19 symptoms in individuals without chronic disease indicates that monitoring and quality-of-life-focused strategies should be implemented not only in patients with chronic respiratory disease but also at the population level.

## Figures and Tables

**Table 1:** Demographic and baseline characteristics of participants.

Variable	Asthma (*n* = 165)	COPD (*n* = 82)	No Chronic Disease (*n* = 431)
Age, mean (IQR)	54 (42–64)	68 (61–74)	38 (27–50)
BMI, mean (SD)	28 (5)	26 (4)	25 (4)
Sex, *n* (%)			
Male	38 (23)	68 (83)	211 (49)
Female	127 (77)	14 (17)	220 (51)
Marital status, *n* (%)			
Living alone	37 (22)	16 (19)	135 (31)
Living with partner	128 (78)	66 (80)	296 (69)
Residence, *n* (%)			
Urban	106 (64)	44 (54)	343 (80)
Rural	59 (36)	38 (46)	86 (20)
Current employment status, *n* (%)			
No	111 (67)	72 (88)	153 (35)
Yes	54 (33)	10 (12)	278 (64)
Change in working environment *,			
*n* (%)			
No	48 (91)	8 (80)	232 (83)
Yes	5 (9)	2 (20)	46 (16)

BMI: body mass index. * Evaluated only among actively working participants.

**Table 2:** Lifestyle and behavioral characteristics during the COVID-19 pandemic.

Variable	Asthma (*n* = 165)	COPD (*n* = 82)	No Chronic Disease (*n* = 431)
Psychological support, *n* (%)			
No	140 (85)	77 (94)	413 (96)
Yes	25 (15)	5 (6)	18 (4)
Sleep changes, *n* (%)			
None	114 (69)	61 (74)	346 (80)
Excessive sleep	15 (9)	1 (1)	12 (3)
Insomnia	13 (8)	9 (11)	23 (5)
Difficulty initiating sleep	12 (7)	5 (6)	25 (6)
Frequent awakenings	11 (7)	6 (7)	25 (6)
Change in tobacco use during the pandemic, *n* (%)			
No	148 (90)	75 (91)	393 (91)
Yes	17 (10)	7 (8)	37 (9)
Smoking behavior, *n* (%)			
Quit	1 (6)	0 (0.)	5 (14)
Reduced	12 (71)	4 (57)	15 (42)
Increased	3 (18)	3 (43)	10 (28)
Initiated during the pandemic	1 (6)	0 (0)	6 (17)
Alcohol use, *n* (%)			
No	158 (96)	74 (90)	336 (78)
Yes	7 (4)	8 (10)	95 (22.)
Change in alcohol use during the pandemic, *n* (%)			
No	161 (98)	78 (95)	415 (96)
Yes	4 (2)	4 (5)	16 (4)

**Table 3:** Physical activity levels assessed by metabolic equivalent of task (MET) scores before and after the pandemic.

Variable	Asthma Mean (SD)	COPD Mean (SD)	No Chronic Disease Mean (SD)	*p* Value
*Pre-pandemic physical activity*				
Vigorous MET	414 (173)	58 (5)	362 (137)	0.146
Moderate MET	489 (114)	189 (84)	390 (80)	0.049
Walking MET	1161 (113)	765 (79)	1377 (126)	<0.001
Sitting MET	10 (4)	12 (5)	10 (4)	<0.001
Total MET	2074 (294)	1024 (157)	2145 (231)	<0.001
*Post-pandemic physical activity*				
Vigorous MET	339 (157)	58 (53)	335 (136)	0.218
Moderate MET	452 (111)	106 (42)	350 (76)	0.010
Walking MET	1027 (110)	733 (94)	1325 (124)	<0.001
Sitting MET	10 (4)	12 (5)	10 (4)	<0.001
Total MET	1828 (278)	910 (145)	2024 (229)	<0.001

**Table 4:** Changes in physical activity categories and mMRC dyspnea scores before and after the pandemic.

Variable	Asthma (*n* = 165)	COPD (*n* = 82)	No Chronic Disease (*n* = 431)	*p* Value
*Pre-pandemic category*				<0.001
Category 1	143 (87)	78 (95)	339 (79)	
Category 2	1 (1)	0 (0)	41 (9)	
Category 3	21 (13)	4 (5)	51 (12)	
*Post-pandemic category*				<0.001
Category 1	147 (89)	79 (96)	348 (81)	
Category 2	1 (1)	0 (0)	38 (9)	
Category 3	17 (10)	3 (4)	45 (10)	
*Change in mMRC score*				0.261
Increased	29 (18)	20 (25)	82 (19)	
Unchanged	131 (80)	56 (71)	343 (80)	
Decreased	3 (2)	3 (4)	5 (1)	

Category 1: <600 MET-min/week (inactive); Category 2: 600–3000 MET-min/week (minimally active); Category 3: >3000 MET-min/week (highly active). MET (metabolic equivalent of task) represents the energy cost of physical activities. mMRC: modified Medical Research Council dyspnea scale. COPD: chronic obstructive pulmonary disease.

**Table 5:** COVID-19 severity and COVID-19-related family loss across study groups.

Variable	Asthma (*n* = 165)	COPD (*n* = 82)	No Chronic Disease (*n* = 431)	*p* Value
*COVID-19 severity, n (%)*				0.000
Outpatient	46 (70)	15 (58)	226 (89)	
Hospitalized	19 (29)	9 (35)	26 (10)	
Intensive care unit	1 (1.5)	2 (8)	3 (1)	
*COVID-19-related family loss, n (%)*				0.008
No	148 (90)	78 (95)	416 (96)	
Yes	16 (10)	4 (5)	15 (3)	

COVID-19 severity was classified according to the highest level of care required during infection. COPD: chronic obstructive pulmonary disease.

## Data Availability

The datasets generated and/or analyzed during the current study are not publicly available due to ethical and privacy restrictions but are available from the corresponding author upon reasonable request.
